# Supplementary material for: Congruence, fossils and the evolutionary tree of rodents and lagomorphs
Source: R Soc Open Sci. 2019 Jul 17;6(7):190387. doi: 10.1098/rsos.190387 (PMC6689570; doi:10.1098/rsos.190387)
Supplement: Systematic and phylogenetic Methods and Tables S1-S5 [file rsos190387supp1.doc]

**Supplementary Information for Asher, Smith, Rankin & Emry, "Congruence, fossils, and the evolutionary tree of rodents and lagomorphs"**

***Royal Society Open Science* 2019**

This supplementary data section includes additional details on systematic and phylogenetic methods, Tables S1 to S5, and Figs. S1 to S7. Graphic documentation of all morphological character states are available in project 2769 on morphobank: http://morphobank.org/permalink/?P2769.

Appendix S1 is an archive with DNA alignment (S1a), indels (S1b), combined data matrix (S1c), and hypothetical ancestors (S1d) in nexus format. Appendix S2 is a spreadsheet with morphological character edits. Appendix S3 is a. spreadsheet with genus-species names, museum numbers, & DNA accession numbers. Appendix S4 provides optimal trees in nexus format. All are available at.datadryad, doi:10.5061/dryad.3840vd7

Supplementary Methods

Recent studies of mammalian phylogenetics strongly support the sister-taxon relationship of rodents and lagomorphs within the Euarchontoglires clade of placental mammals ([[1]](#endnote-2),[[2]](#endnote-3)). Relationships within Rodentia are also now reasonably well understood ([[3]](#endnote-4)). These studies have identified three major clades: squirrel-related, mouse-related, and guinea pig-related. The latter was named Ctenohystrica by Huchon et al. ([[4]](#endnote-5)); the names Sciuromorpha and Myomorpha are available ([[5]](#endnote-6)) for squirrel- and mouse-related clades, respectively. Fabre et al. ([[6]](#endnote-7)) used Ctenohystrica but did not name high-level taxa for mouse- or squirrel-related clades. In fig. 1 of their paper, Fabre et al. (Error: Reference source not found) designated three clades within Myomorpha: Castorimorpha (for *Castor* plus geomyoids), Anomaluromorpha (for *Anomalurus* and *Pedetes*) and Myodonta (for muroids and dipodids). This terminology is compatible with ours; to minimize jargon we do not coin any new names ([[7]](#endnote-8)), but use the existing names Myomorpha for the mouse-related and Sciuromorpha for the squirrel-related clades.

Churakov et al. ([[8]](#endnote-9)) found support for a Myomorpha-Ctenohystrica clade to the exclusion of Sciuromorpha. Within the latter, glirids were sister to an *Aplodontia*-sciurid clade. Ctenohystrica consisted of a *Laonastes* (Diatomyidae ([[9]](#endnote-10)))*-*ctenodactylid clade as sister to hystricognaths; Myomorpha consisted of geomyoids, *Castor*, a *Pedetes-Anomalurus* clade, and a dipodid, spalacid, and cricetid-murid clade. Rodriguez-Prieto et al. ([[10]](#endnote-11)) figured a phylogeny estimated from 10 genomic introns across 14 rodents, rooted at *H. sapiens*. Their likelihood topology (their fig. 3A) was completely congruent with the phylogeny of Churakov et al. (Error: Reference source not found); their Bayesian topology (fig. 3B in (Error: Reference source not found)) was nearly congruent except for the root position, showing Myomorpha rather than Sciuromorpha at the base of Rodentia. The consensus topology derived from microRNAs (Error: Reference source not found), SINEs (Error: Reference source not found), introns (Error: Reference source not found) and RGCs ([[11]](#endnote-12)) is independent of the morphological data and DNA sequence alignment sampled in this study; we therefore used it (main text g. S13) as a baseline to infer performance of our data partitions and to generate character states for our hypothetical ancestors. Polytomies in . S1 3 reflect uncertainty, either due to conflict (e.g., the rodent root) or due to inclusion of low-level taxa not sampled by the studies of independent datasets cited above but widely accepted to be close relatives of the species that are sampled for SINEs, microRNAs, introns, and/or RGCs. This includes taxa such as *Gerbillus* and *Massoutiera*; while not sampled by Churakov et al. (Error: Reference source not found), they are widely regarded as (respectively) a gerbilline murid along with *Meriones* and *Dipodillus*, and a ctenodactylid along with *Ctenodactylus* ([[12]](#endnote-13)), species which are sampled for genomic data independent of our dataset.

Taxon and character sampling. We did not include genes that were unknown for more than two geomyoids or over 50% of all taxa in our sample. We sampled only contiguous sequences that were easily alignable to positively identified orthologs in the "gene" and/or "definition" fields on Genbank (https://www.ncbi.nlm.nih.gov/nuccore?db=nucleotide), and which exhibited sequence overlap among key taxonomic groups. For example, RAG1 sequences are known for *Echimys*, *Myoxus*, and *Thryonomys*, but these align beyond the 3' end of samples available for our other taxa and so were excluded from this study. Our sample of RAG1 resulted in an alignment of just under 1kb of overlapping DNA among taxa such as *Dipodomys* (JN414968) and *Didelphis* (DQ865890). In all cases, overlap of sequences across taxa within each gene had to be sufficient to enable an initial parsimony (MP) analysis to complete at least 100 random addition sequence replicates and result in one or few optimal trees in PAUP ([[13]](#endnote-14)), typically within a few seconds of computer time.

We used Clustal Omega (https://www.ebi.ac.uk/Tools/msa/clustalo/) and Muscle (https://www.ebi.ac.uk/Tools/msa/muscle/) packages for initial alignments using default parameters. We then visually inspected alignments with Mesquite (v. 3.51, ([[14]](#endnote-15))) and undertook regional re-alignments with the Opal package (v2.1, ([[15]](#endnote-16))) to preserve reading frames for coding loci and verify sequence homology. After alignment, isolated leading and trailing sequences were deleted. Several regions of *12s* and *16s* rRNA and *A2AB* were too variable to establish homology and were omitted from our final alignment. Remaining gap characters were treated as "missing" but the phylogenetic information they represent as insertions or deletions ("indels") was distilled into a separate matrix for each gene. Overlapping indels of varying sizes were coded as a single character, except in some cases where part of an indel overlapped with those in other taxa of variable lengths. In such cases we coded indels according to each instance of overlap that was shared with other taxa. State "1" represents insertions and "0" deletions. We identified each indel character by the number of the first or last aligned indel site common to all taxa that possess it, as identified in supplementary appendix S1.

Two of our loci (*IRBP* and *cytB*) were available for all 60 of our extant terminals. Three (*12S*, *COX1*, *GHR*) were known for 57-59 terminals, six (*16S*, *vWF*, *RAG1*, *BRCA1*, *CNR1*, *A2AB*) for 49-52, and three (*COX2*, *COX3*, *ATP7A*) for 39-42. Supplementary appendix S2 provides further details on our DNA sample, including accession numbers and taxon names. Sequences from *12S* rRNA, *COX2* and *COX3* were initially derived from the alignment of Fabre et al. (Error: Reference source not found). In cases where a more complete sequence for a given taxon was publicly available, this replaced sequences used by Fabre et al. (Error: Reference source not found), as indicated in supplementary appendix S2. In some cases we substituted shorter sequences (e.g., *12S* rRNA for *Arvicola "terrestris"* (= *A. amphibius*) EF027242.1) when a longer sequence (e.g., AY948543.1) proved difficult to align. Sequences of *BRCA1* were chosen to maximize overlap with BRCA1 available for *Dipodomys heermanni* exon 11 (JN414201.1). In one case, we deleted a unique, 57bp insertion ("ccagagaaccacaaataacacaagagcttgccagagaaccacaaataacacaacagc") from *Glaucomys* *BRCA1* (AF284003) starting at site 642 of the genbank entry and position 525 of our *BRCA1* alignment.

We concatenated sequences at genus level in order to maximize coverage across our samples for morphological and DNA-indel data, and followed Fabre et al. (Error: Reference source not found) and Helgen et al. ([[16]](#endnote-17)) to ensure each of our genus-level terminals comprised monophyletic taxa, and Wilson & Reeder (Error: Reference source not found) as a baseline for species-level taxonomy. For example, *Marmota* included *M. bobak* for morphology, *M. monax, M. marmota,* and *M. himalayana* for DNA+indels. *Arvicanthis "somalicus"* and *A. "variegatus"* are junior synonyms of, respectively, *A. neumanni* (sampled for DNA) and *A. niloticus* (sampled for morphology and DNA). Morphological data for *Gerbillus* were derived in part from a specimen of "*Dipodillus*" (UMZC E.1971, genus *Gerbillus*, subgenus *Hendecapleura*) *watersi*, collected from Merowe in today's Sudan near the type locality of *Gerbillus watersi* (Shendi, Sudan). Morphological data for *Capromys* were derived from a specimen of *Geocapromys browni* (UMZC E.3379), often regarded in past literature as a subgenus within *Capromys* (Error: Reference source not found).

DNA partitions and models of sequence evolution. For our Bayesian phylogenetic analyses, we used PartitionFinder2 ([[17]](#endnote-18)) and PhyML ([[18]](#endnote-19)) to identify an optimal partition scheme and models for our sequence data. We used BIC (Bayesian Information Criterion) to identify optimal DNA character partitions and substitution models (Table S1). We treated our 219 morphological characters as a single partition using the "standard discrete" model ([[19]](#endnote-20)) with gamma-distributed rate variation (p. 975 in Ronquist et al. ([[20]](#endnote-21))); for indels we used the restriction site model in MrBayes. Both assumed that only variable characters were included ("lset coding=variable"). The MPI version of MrBayes 3.2.7 ([[21]](#endnote-22),[[22]](#endnote-23)) on the University of Cambridge HPC cluster (with 32 cores per processor) enabled us to run our dataset using 8 runs with 4 chains each, with 16-35 million ("M") generations, sampling every 1000. The optimal topology, branch lengths, and posterior probabilities are derived from a majority rule consensus across all sampled, post-burnin trees from all runs, with burnin defined as a global application of 50% across all runs (Table S4A). We also explored burnin values independently for each run using Tracer 1.6 ([[23]](#endnote-24)), in increments of 0.5M generations, and picked values which maximized the effective sample size of log-likelihood scores for that run while keeping at least 10M of the 35M generations for each of the 102 taxon runs and 5M of the 30M generations for each of the 60 taxon runs (Table S4B).

MP search strategies. For parsimony (MP) searches, we used TNT ([[24]](#endnote-25)) and PAUP 4.0a (Error: Reference source not found). The following TNT commands typically yielded optimal results in the shortest time: "hold 1000; xmult=hits 15 rss fuse 5 drift 10 ratchet 20; bbreak=tbr", increasing to "hold 10000" in cases where the tree buffer overflowed. MP searches in TNT enabled us to undertake several thousand distinct phylogenetic analyses of our dataset, each of which required seconds or minutes to complete, rather than hours or days often required by probabilistic optimality criteria. To explore the effect of character weighting on congruence with the well-corroborated tree, we used a range of concavity (or "*k*") values for implied weighting as a function of homoplasy ([[25]](#endnote-26)) with the command "piwe=*k*" where *k* = 2, 4, 6, 8, 10, 12, 14, 16, 24, 32, 48, 64, 128, 256, or 512. We saved optimal topologies in newick format ("taxname =; export - filename.tre") and calculated strict and majority-rules consensuses with "nelsen*" and "majority*" (respectively) as the last two newick trees written to each treefile.

**Table S1.** Optimal partition scheme identified by PartitionFinder2 (Error: Reference source not found) using BIC to distinguish among partitioning schemes and a greedy search among models available in MrBayes (Error: Reference source not found). Supplementary file S1 gives our alignment; S2 gives our genbank accession numbers; P1, P2, and P3 refer to first, second, and third codon positions, respectively.

| Best Model | # sites | Partition names |
| --- | --- | --- |
| GTR+G | 1073 | cnr1P3, rag1P3, a2abP3 |
| SYM+I+G | 1087 | rag1P1, a2abP2, a2abP1 |
| GTR+I+G | 1939 | rag1P2, atp7aP1, ghrP2, atp7aP2, vwfP2, irbpP2 |
| GTR+I+G | 798 | brca1P3, atp7aP3 |
| GTR+I+G | 1139 | brca1P2, brca1P1 |
| SYM+I+G | 366 | cnr1P1 |
| HKY+I+G | 864 | co1P2, cnr1P2 |
| GTR+I+G | 1157 | ghrP1, vwfP1, irbpP1 |
| K80+G | 311 | ghrP3 |
| GTR+G | 845 | vwfP3, irbpP3 |
| SYM+I+G | 498 | co1P1 |
| GTR+I+G | 726 | co2P3, co1P3 |
| GTR+I+G | 870 | co3P1, co2P1, cytbP1 |
| GTR+I+G | 869 | co2P2, cytbP2, co3P2 |
| GTR+I+G | 641 | cytbP3, co3P3 |
| GTR+I+G | 2224 | 12s, 16s |

**Table S2.** Proportion of shared quartets between strict-consensus MP topologies of extant taxa relative to the well-corroborated tree (Fig. S1). Each row corresponds to the mean proportion of shared quartets of 25 phylogenetic analyses with and 25 without morphological data, varying number of subsampled DNA-indel sites from 312 to 14036. Two-way, non-parametric Wilcoxon and Kolmogorov-Smirnov (KS) p values represent rejected (bold) or accepted (plain text) null hypothesis that mean proportion of shared quartets does not significantly change with addition of 219 morphological characters. As sample length increases, ties become more frequent and potentially make the KS p values more appropriate.

| sample length in bp | Shared quartets DNA+indel+morphology | Shared quartets DNA+indel | KS p value | Wilcoxon p value |
| --- | --- | --- | --- | --- |
| 312 | 0.7405949 | 0.5530574 | **0** | **0** |
| 780 | 0.8229624 | 0.7163124 | **0.0008** | **0.0004** |
| 1560 | 0.8859752 | 0.770616 | **0.0008** | **0.0008** |
| 3119 | 0.9304776 | 0.8900847 | 0.1545 | 0.1595 |
| 4679 | 0.9595089 | 0.9327678 | **0.0366** | 0.0711 |
| 6238 | 0.9830731 | 0.9592549 | **0.0366** | 0.1096 |
| 7798 | 0.9908604 | 0.9755791 | **0.0158** | **0.0119** |
| 9358 | 0.9951592 | 0.9794198 | 0.1545 | **0.0219** |
| 10917 | 0.9967695 | 0.9895081 | 0.1545 | 0.0535 |
| 12477 | 0.999183 | 0.9960552 | 0.9062 | 0.1371 |
| 14036 | 0.9995759 | 0.994661 | 0.9062 | **0.0412** |

**Table S3.** Summary of optimal MP trees. Each tree is available in nexus format as part of our supplementary appendix S4, following the names in the first column. "Hyp ancs" = hypothetical ancestors, "*k*" = MP implied weighting concavity values that recovered the most shared partitions, "# trees" = number of shortest MP trees.

| name | weights | data | taxa | # trees | steps |
| --- | --- | --- | --- | --- | --- |
| MPmorphAncs | equal | morphology | 60 extant, 29 hyp ancs | 128 | 1603 |
| MPmorphAll | equal | morphology | 60 extant, 42 extinct | 140 | 2039 |
| MPmorphExt | equal | morphology | 60 extant | 60 | 1511 |
| MPmorphDNAextant | equal | morphology, DNA+indels | 60 extant | 1 | 65092 |
| MPall | equal | morphology, DNA+indels | 60 extant, 42 extinct | 56 | 65599 |
| MPdnaExt | equal | DNA+indels | 60 extant | 1 | 63437 |
| morphAllK24 | *k* = 24 | morphology | 60 extant, 42 extinct | 1 | 49.50103 |
| morphExtK2 | *k* = 2 | morphology | 60 extant | 1 | 129.98922 |
| CombAllK2 | *k* = 2 | morphology, DNA+indels | 60 extant, 42 extinct | 1 | 1410.38448 |

**Table S4.**

A) Optimal burnin values and indicators of convergence for Bayesian phylogenetic analyses. All analyses consisted of 8 runs of 4 chains each sampled every 1000 generations. "Burn-in" = number of generations of mcmc output discarded as outside region of stationarity, "Mgen" = millions of generations, ESS = effective sample size, lnL = log likelihood, SDSF = standard deviation of split frequencies. Each majority rule, post burn-in topology is available in nexus format as part of our supplementary appendix S4, following the names in the first column.

| name | data | taxa | Mgen | burn-in | optimal lnL | SDSF | ESS lnL |
| --- | --- | --- | --- | --- | --- | --- | --- |
| BayesAll_tracer | morphology, DNA+indels | 60 extant, 42 extinct | 35 | Tracer (see B) | -272708.2101 | - | 545 |
| BayesAll_50 | morphology, DNA+indels | 60 extant, 42 extinct | 35 | global 50% | -272709.5483 | 0.027415 | 398 |
| BayesDNAext | DNA+indels | 60 extant | 30 | global 50% | -264429.3274 | 0.001026 | 794 |
| BayesMorphExt | morphology | 60 extant | 16 | global 50% | -5553.084 | 0.009924 | 18776 |
| BayesMorphAll | morphology | 60 extant, 42 extinct | 20 | global 50% | -7753.152 | 0.043537 | 12720 |

B) Custom burnin values for combined DNA-indel-morphology for extant taxa and fossils (35 million or "M" generations, columns 2, 3) and DNA-indel for extant taxa only (30M generations, columns 4, 5) Bayesian analyses as determined by Tracer v1.6 (Error: Reference source not found).

| run | 60 extant, 42 extinct burn-in Mgen | 60 extant, 42 extinct ESS lnL | 60 extant burn-in Mgen | 60 extant ESS lnL |
| --- | --- | --- | --- | --- |
| run1.p | 17.5 | 245 | 25 | 987 |
| run2.p | 27.5 | 357 | 11 | 449 |
| run3.p | 20 | 788 | 23 | 1847 |
| run4.p | 25 | 151 | 13.5 | 3251 |
| run5.p | 22 | 932 | 24.5 | 1129 |
| run6.p | 23 | 476 | 21.5 | 1137 |
| run7.p | 26.5 | 531 | 14 | 3943 |
| run8.p | 14 | 677 | 17.5 | 1861 |
| combined |  | 545 |  | 1624 |

Table S5: Supplementary R script

#initial analyses derived from script written by Seraina Klopfstein (https://www.nmbs.ch/home/museum/team/seraina-klopfstein.html) and modified by Robert Asher (http://people.ds.cam.ac.uk/rja58/). Script below is by Martin Smith (https://community.dur.ac.uk/martin.smith/)

# Load required libraries

library("ape")

library("Quartet")

# Load reference tree

referenceTree <- read.tree(file.choose())

referenceTips <- referenceTree$tip.label

# Function to prune non-shared taxa and generate consensus

PrunedConsensus <- function (file) {

lines <- readLines(file, warn=FALSE)[-(1:3)]

nLines <- length(lines)

TNT_EXTRAS <- 2L # the last two trees in each file are generated by nelsen* and majority*

trees <- read.tree(text=lines[seq_len(nLines / 2L - 1L - TNT_EXTRAS) * 2L])

if (class(trees) == 'phylo') trees <- structure(list(trees), class='multiPhylo')

# Return:

consensus(lapply(trees, function (cf) {

drop.tip(cf, setdiff(cf$tip.label, referenceTips))

}))

}

testFiles <- choose.files()

testTrees <- lapply(testFiles, PrunedConsensus)

testQuartets <- SharedQuartetStatus(testTrees, referenceTree)

testQD <- QuartetDivergence(testQuartets, similarity=FALSE)

testSQ <- testQuartets[, 's', drop=FALSE] / rowSums(testQuartets[, c('s', 'd', 'r2'), drop=FALSE])

write(testQD, file="QuartetDivergence.txt")

write(testSQ, file="SharedQuartets.txt")

**Supplementary Information** **References**

1. Tarver JE, et al. (2016) The Interrelationships of Placental Mammals and the Limits of Phylogenetic Inference. *Genome Biol Evol* 8(2):330–344. [↑](#endnote-ref-2)
2. Esselstyn JA, Oliveros CH, Swanson MT, Faircloth BC (2017) Investigating Difficult Nodes in the Placental Mammal Tree with Expanded Taxon Sampling and Thousands of Ultraconserved Elements. *Genome Biol Evol* 9(9):2308–2321. [↑](#endnote-ref-3)
3. Cox PG, Hautier L (2015) *Evolution of the rodents: Advances in Phylogeny, Functional morphology and Development*. (Cambridge University Press). [↑](#endnote-ref-4)
4. Huchon D, Catzeflis FM, Douzery EJP (2000) Variance of molecular datings, evolution of rodents and the phylogenetic affinities between Ctenodactylidae and Hystricognathi. *Proceedings of the Royal Society of London B: Biological Sciences* **267,** 393–402. [↑](#endnote-ref-5)
5. Brandt JF (1855) *Beiträge zur näheren Kenntniss der Säuge-Thiere Russlands: Mit 19 Tafeln.(Aus den Mém. Mathémat., phys. Et nat., Tom. VII. Besonders abgedruckt.)*. (Kaiserl Academ d Wiss). [↑](#endnote-ref-6)
6. Fabre P-H, Hautier L, Dimitrov D, Douzery P, Emmanuel J (2012) A glimpse on the pattern of rodent diversification: a phylogenetic approach. *BMC Evol Biol* 12(1):88. [↑](#endnote-ref-7)
7. Asher RJ, Helgen KM (2010) Nomenclature and placental mammal phylogeny. *BMC Evolutionary Biology* **10,** 102. [↑](#endnote-ref-8)
8. Churakov G, et al. (2010) Rodent evolution: back to the root. *Mol Biol Evol* 27(6):1315–1326. [↑](#endnote-ref-9)
9. Dawson MR, Marivaux L, Li C, Beard KC, Métais G. (2006) *Laonastes* and the "Lazarus effect" in recent mammals. *Science* **311,** 1456–1458. [↑](#endnote-ref-10)
10. Rodriguez-Prieto A, Igea J, Castresana J (2014) Development of rapidly evolving intron markers to estimate multilocus species trees of rodents. *PLoS One* 9(5):e96032. [↑](#endnote-ref-11)
11. Mason VC, et al. (2016) Genomic analysis reveals hidden biodiversity within colugos, the sister group to primates. *Sci Adv* 2(8):e1600633. [↑](#endnote-ref-12)
12. Wilson DE, Reeder DM (2005) *Mammal species of the world: a taxonomic and geographic reference*. (JHU Press). [↑](#endnote-ref-13)
13. Swofford DL (2002) PAUP*: phylogenetic analysis using parsimony (* and other methods) 4.0a. Sunderland, MA. [↑](#endnote-ref-14)
14. Maddison WP, Maddison DR (2015) Mesquite: a modular system for evolutionary analysis. [↑](#endnote-ref-15)
15. Wheeler TJ, Kececioglu JD (2007) Multiple alignment by aligning alignments. *Bioinformatics* **23,** i559--i568. [↑](#endnote-ref-16)
16. Helgen KM, Cole FR, Helgen LE, Wilson DE (2009) Generic revision in the Holarctic ground squirrel genus Spermophilus. *Journal of Mammalogy* **90,** 270–305. [↑](#endnote-ref-17)
17. Lanfear R, Frandsen PB, Wright AM, Senfeld T, Calcott B (2016) PartitionFinder 2: new methods for selecting partitioned models of evolution for molecular and morphological phylogenetic analyses. *Molecular Biology and Evolution* **34,** 772–773. [↑](#endnote-ref-18)
18. Guindon S. *et al.* (2010) New algorithms and methods to estimate maximum-likelihood phylogenies: assessing the performance of PhyML 3.0. *Systematic biology* **59,** 307–321. [↑](#endnote-ref-19)
19. Lewis PO (2001) A likelihood approach to estimating phylogeny from discrete morphological character data. *Syst Biol* 50(6):913–925. [↑](#endnote-ref-20)
20. Ronquist F. *et al.* (2012) A total-evidence approach to dating with fossils, applied to the early radiation of the Hymenoptera. *Systematic Biology* **61,** 973–999. [↑](#endnote-ref-21)
21. Ronquist F *et al.* (2012) MrBayes 3.2: efficient Bayesian phylogenetic inference and model choice across a large model space. *Systematic biology* **61,** 539–542. [↑](#endnote-ref-22)
22. Altekar G, Dwarkadas S, Huelsenbeck JP, Ronquist F (2004) Parallel metropolis coupled Markov chain Monte Carlo for Bayesian phylogenetic inference. *Bioinformatics* **20,** 407–415. [↑](#endnote-ref-23)
23. Rambaut A, Drummond AJ, Suchard M (2013) Tracer v1. 6—MCMC trace analysis package. *Institute of Evolutionary Biology, University of Edinburgh, UK*. [↑](#endnote-ref-24)
24. Goloboff PA, Catalano SA (2016) TNT version 1.5, including a full implementation of phylogenetic morphometrics. *Cladistics* 32(3):221–238. [↑](#endnote-ref-25)
25. Goloboff PA, Carpenter JM, Arias JS, Esquivel DRM (2008) Weighting against homoplasy improves phylogenetic analysis of morphological data sets. *Cladistics* 24(5):758–773. [↑](#endnote-ref-26)
