# Supplementary material for: Congruence, fossils and the evolutionary tree of rodents and lagomorphs
Source: R Soc Open Sci. 2019 Jul 17;6(7):190387. doi: 10.1098/rsos.190387 (PMC6689570; doi:10.1098/rsos.190387)

Fig. S1. Mean number of partitions shared with well-corroborated tree (Y-axis) with N added hypothetical ancestors (X-axis) estimated with acctran (black triangles) and deltran (red circles) to MP morphology analyses of 60 extant taxa. Polygons represent mean shared partitions across 25 replicates per N added taxa of randomly chosen ancestors, vertical error bars 1/2 standard deviation on either side of mean. X-axis values arbitrarily separated by 0.5 to minimize overlap of polygons and lines.

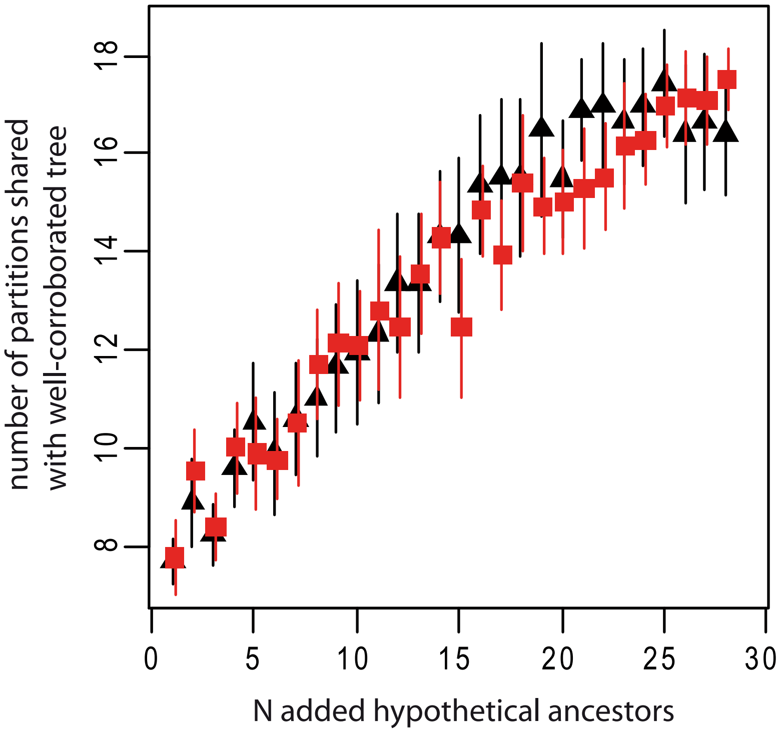

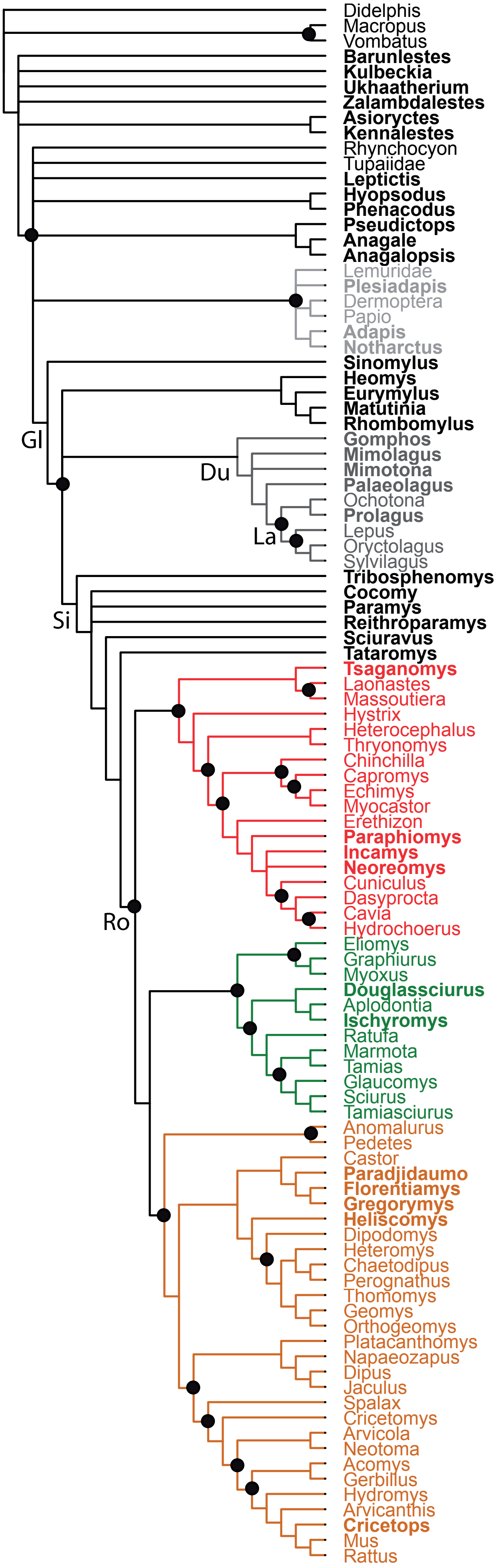

Fig. S2. Strict consensus of 56 MPTs, 65599 steps, derived from TNT search of 219 morphological and 15595 DNA-indel characters, treating all changes equally. Du = Duplicidentata, Gl = Glires, Ro = Rodentia, Si = Simplicidentata. Nodes shared with well-corroborated tree are shown with black circles. Primates-Dermoptera are shown in light gray, Duplicidentata (including Lagomorpha) in dark gray, Sciuromorpha in green, Myomorpha in orange, and Ctenohystrica in red. ; branch lengths are arbitrary.

Fig. S3. A) Majority rule consensus of 15,000 post-burnin (50% of 30G generations) Bayesian topologies based on DNA-indels for extant taxa. All nodes have a posterior probability of 1 except where indicated. Burnin values defined in Table S4 yield the same topology and posterior probabilities to the nearest 0.05. Colors are as in Fig. S2. B) Single MPT of 63437 steps for same dataset, all changes equal. Oblique lines represent clades that differ from Bayesian topology (A). Branch lengths within Placentalia in A correspond to scale at bottom left; non-placental (A) and MP branch lengths (B) are arbitrary.

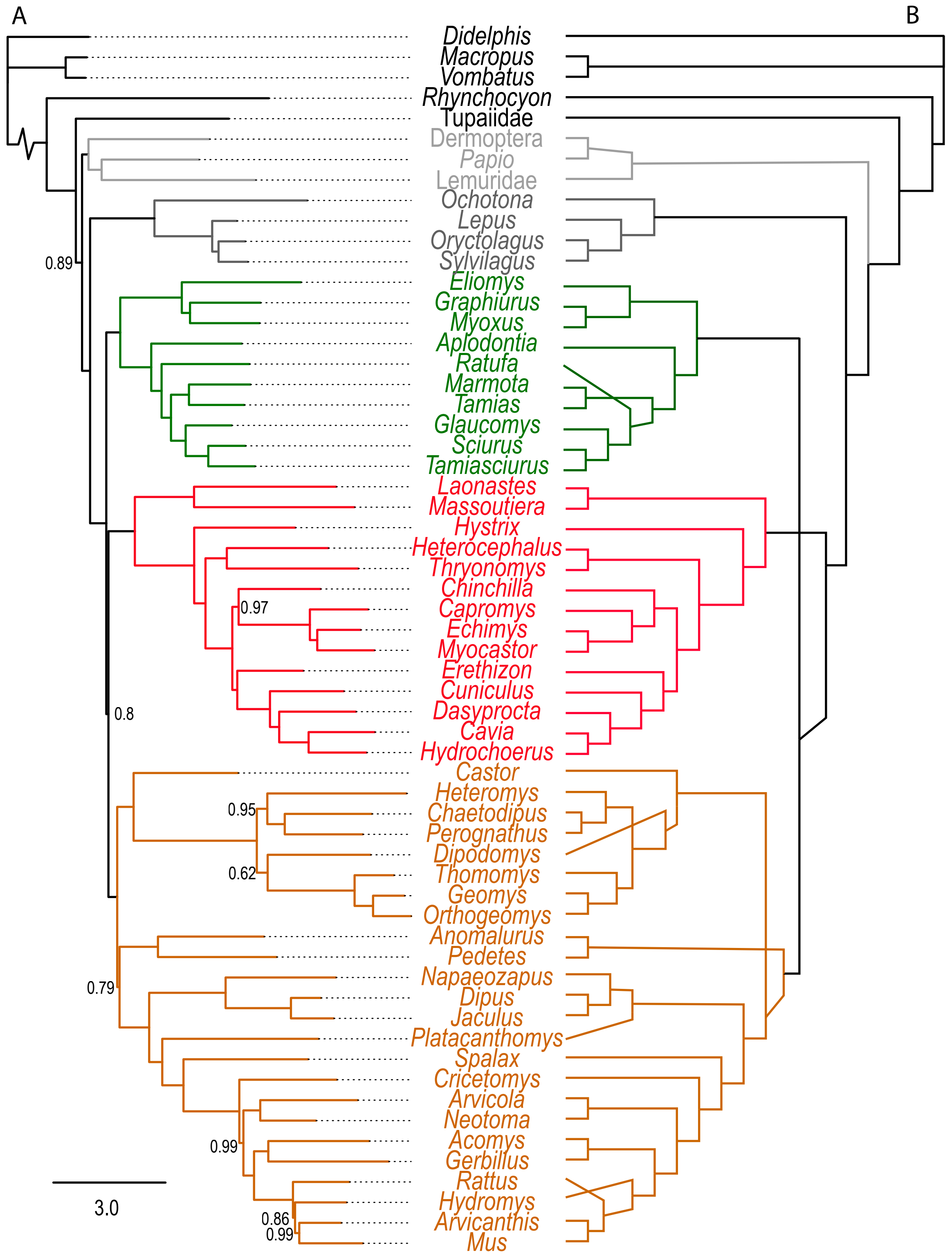

Fig. S4. Number of partitions shared with well corroborated tree (Y-axis) resulting from addition of 1-18 added extant taxa (X-axis, open triangles), fossils (yellow circles), and hypothetical ancestors (green squares) to 41-taxon dataset, each showing the mean (polygon) and one standard deviation (vertical lines) across 18 replicates per number of added taxa on X-axis. X-axis values are separated arbitrarily by 0.1 to minimize overlap of polygons and lines

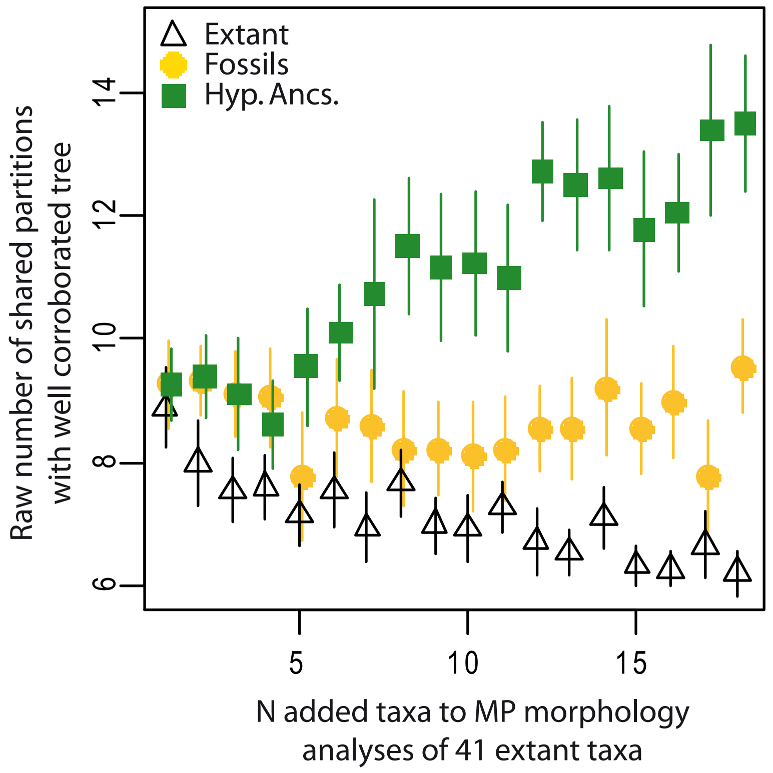

Fig. S5. MP strict consensus topologies derived from equally weighted, morphological data for A) living taxa (60 trees, 1511 steps), B) living plus 42 fossils (140 trees, 2039 steps), and C) living taxa plus 29 hypothetical ancestors (128 trees, 1603 steps). "Anc" corresponds to internal nodes of well-corroborated tree optimized for morphological data treated as terminals (see main text). Nodes shared with well-corroborated tree (as measured with shared partitions in Mesquite) are shown with black circles. Colors are as in Fig. S2; fossils are in bold; branch lengths are arbitrary.

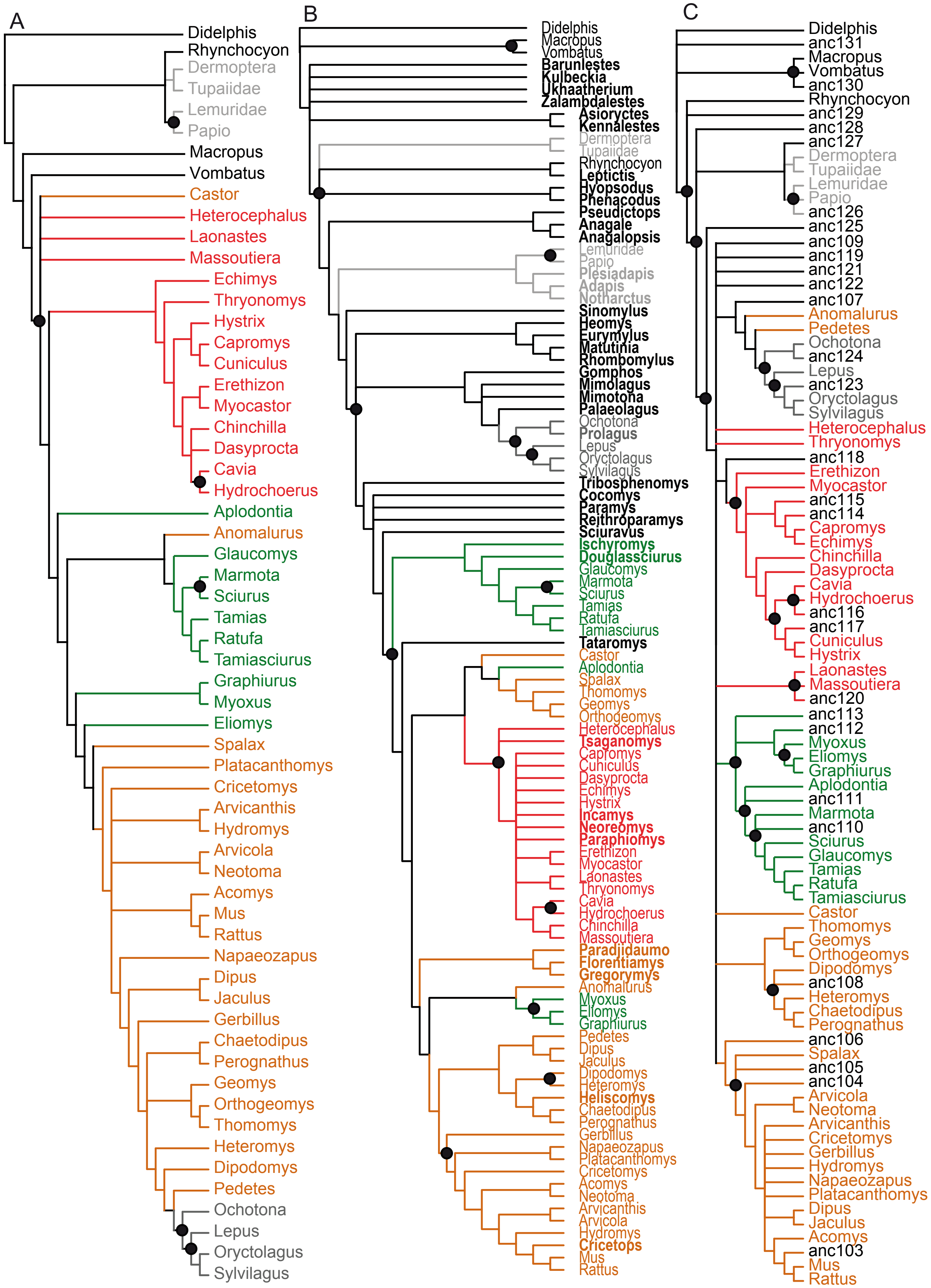

Fig. S6. Number of partitions shared with well corroborated tree (Y-axis) of optimal MP trees with implied weights in TNT with k = 2, 4, 6, 8, 10, 12, 14, 16, 24, 32, 48, 64, 128, 256, 512, and equal weights (X-axis). Polygons connected with lines show morphological data for extant taxa (red triangles), morphological data for extant taxa plus fossils (black circles), DNA+indels for extant taxa (light blue circles) and DNA+indels+morphology for extant taxa plus fossils (dark blue crosses). Arrow indicates break in X-axis scale.

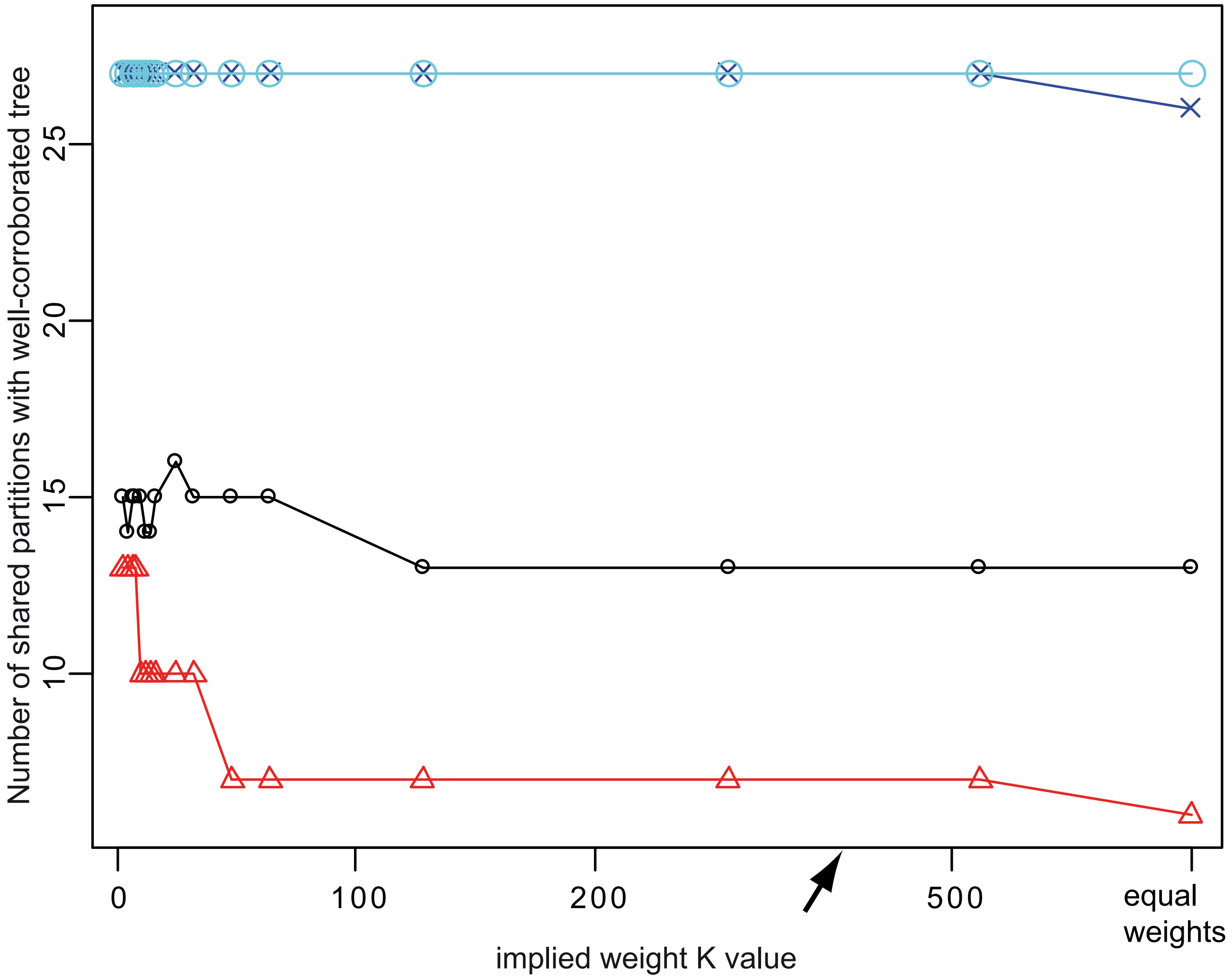

Fig. S7. Number of partitions shared (squares, right Y axis) and Robinson-Foulds distances standardized by proportion resolved (circles, left Y axis) relative to well-corroborated tree. X-axis shows varying samples from our morphology+DNA+indel dataset and analysis parameters. These indices behave similarly as (respectively) shared quartets and normalized symmetric difference, as discussed in the text. Parameters for each analysis on the X-axis are as follows:

data, d (dna+indels), "c" (combined morphology+dna+indels), or "m" (morphology);

taxa, e (60 extant taxa), "f" (42 fossils and 60 extant taxa), or "a" (29 hypothetical ancestors and 60 extant taxa);

optim or optimality criterion, "b" Bayesian, "∞" = equally weighted parsimony,
"2" and "24" = k concavity integers used for parsimony implied weights that yielded the highest number of shared partitions.

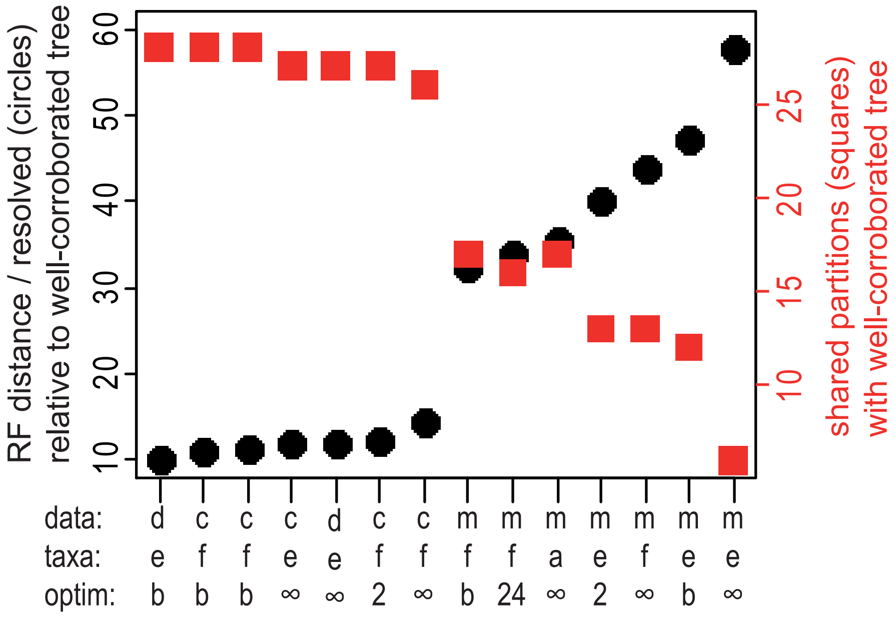

Supplement: Figures S1-S7 [file rsos190387supp2.pdf]
